# Accessing depth-resolved high spatial frequency content from the optical coherence tomography signal

**DOI:** 10.1038/s41598-021-96619-7

**Published:** 2021-08-24

**Authors:** Sergey Alexandrov, Anand Arangath, Yi Zhou, Mary Murphy, Niamh Duffy, Kai Neuhaus, Georgina Shaw, Ryan McAuley, Martin Leahy

**Affiliations:** 1 National University of Ireland, National Biophotonics and Imaging Platform, School of Physics, Tissue Optics and Microcirculation Imaging Group, Galway, H91 TK33 Ireland; 2 Regenerative Medicine Institute, School of Medicine, National University of Ireland, Galway, Ireland; 3 Institute of Photonic Sciences (ICFO), Barcelona, Spain

**Keywords:** Optical techniques, Imaging and sensing, Imaging techniques, Optical imaging

## Abstract

Optical coherence tomography (OCT) is a rapidly evolving technology with a broad range of applications, including biomedical imaging and diagnosis. Conventional intensity-based OCT provides depth-resolved imaging with a typical resolution and sensitivity to structural alterations of about 5–10 microns. It would be desirable for functional biological imaging to detect smaller features in tissues due to the nature of pathological processes. In this article, we perform the analysis of the spatial frequency content of the OCT signal based on scattering theory. We demonstrate that the OCT signal, even at limited spectral bandwidth, contains information about high spatial frequencies present in the object which relates to the small, sub-wavelength size structures. Experimental single frame imaging of phantoms with well-known sub-micron internal structures confirms the theory. Examples of visualization of the nanoscale structural changes within mesenchymal stem cells (MSC), which are invisible using conventional OCT, are also shown. Presented results provide a theoretical and experimental basis for the extraction of high spatial frequency information to substantially improve the sensitivity of OCT to structural alterations at clinically relevant depths.

## Introduction

Since its invention in the early 1990s^1^, the motivating factor behind the development of OCT tools and techniques has been to enable in situ imaging of tissue microstructures with a resolution approaching that of histology, without the need for tissue excision. OCT provides unique depth-resolved morphologic and functional information, which helps with the diagnosis and monitoring of diseases^2–12^. Other methods, such as phase-sensitive OCT or speckle OCT, can detect changes within the sample smaller than the size of the coherent gate^13,14^.

The invention of Fourier domain OCT (FDOCT), including spectral-domain OCT (SDOCT) and swept-source OCT (SSOCT), significantly improved the speed and sensitivity in comparison with time-domain OCT (TDOCT)^15^. The resolution and sensitivity of intensity-based OCT systems to structural changes depends on the spectral bandwidth and remains on the micrometer scale. The physical basis and corresponding image reconstruction procedure for FDOCT are different from TDOCT and is based on the inverse Fourier transform. For the reconstruction of the depth profile of an object, it is necessary to have the correct Fourier spectrum of the axial spatial frequencies of the object. Formation of a spectrum and its spatial frequency content in reflection configuration was considered in the literature^16–20^ but usually not directly related to OCT.

In the current paper, we analyze the spatial frequency content of the OCT signal based on general scattering theory^16^ and show, theoretically and experimentally, that the information about high spatial frequencies of the object, that relates to its small, sub-wavelength size structure, is present in the OCT signal. This information is detected using FDOCT; the recorded spectral interference signal is a rescaled complex Fourier spectrum of the axial high spatial frequencies of the object. Finally, a solution is presented to keep and utilize high spatial frequency information from just a single B-frames. Moreover, the visualization of the sub-wavelength structures, which would be invisible with conventional OCT, is demonstrated experimentally using phantoms with known internal structure, and on mesenchymal stem cells (MSCs).

## Results

### Information content of the OCT signal

The theoretical analysis of the scattered field from an object in reflection configuration is presented in the Supplementary Information. There it is shown that high spatial frequency information is present in the reflected waves and that this information is spectrally encoded. OCT is typically a one-dimensional solution of the inverse scattering problem and usually works using low NA objective lenses. The collected axial spatial frequency can be expressed as

1 $$v_{z} = {{n\left( {\cos \theta + \cos \alpha } \right)} \mathord{\left/ {\vphantom {{n\left( {\cos \theta + \cos \alpha } \right)} \lambda }} \right. \kern-\nulldelimiterspace} \lambda },$$

where *α* and *θ* are the scattering and illumination angles. For reflection configuration at small illumination and collection angles *θ* ≈ 0, *α* ≈ 0, which is typical for OCT, *k*_*0z*_* ≈ k* and Eq. (1) can be simplified as

2 $$v_{z} = \frac{2n}{\lambda }.$$

In this case, only axial spatial frequencies are presented in the collected scattered wave and all accessible spatial frequencies are along the central green arrow in Supplementary Fig. S1b. Thus, from Supplementary Fig. S1b and Eqs. (1, 2), we can see that within moderate NA of the objective lens and limited spectral bandwidth, there is a one-to-one correspondence between axial spatial frequency and wavelength. A spectral encoding of spatial frequency (SESF) approach uses this to encode axial spatial frequency through spectral diversity, translate spatial information from the Fourier domain into the image domain as wavelengths, independently of the resolution of the optical imaging system, and map to each pixel of the 2D image^17,21–25^. The uncertainties in spatial frequencies/periods depending on NA of the objective lens were analysed previously^17,21^. It is essential that in reflection configuration, the high spatial frequencies, which correspond to small, sub-wavelength size structure, are captured. For example, even for a wavelength of 1300 nm, the corresponding spatial frequency, according to Eq. (2), is *v*_*z*_ = 1538 l/mm for *n* = 1, and the corresponding spatial period of the object’s structure is 650 nm. At the same time, the axial spatial frequency profiles are ultra-sensitive to structural changes^17,21–25^.

To collect the complex amplitudes of the spatial frequency components and form $\tilde{F }\left(\mathbf{K}\right)$ (Supplementary Eq. S7 in Supplementary Information), the interference signal using a reference wave can be formed^1^. FDOCT permits detecting the spectral interference signal where we have a one-to-one correspondence between wavelengths and complex amplitudes of the axial spatial frequency components *v*_*z*_. After that, the spectral interference FDOCT signal can be easily rescaled to complex amplitudes of the axial spatial frequencies, representing the Fourier spectrum of the axial profile of the object, using Eq. (2), meaning that axial spatial frequencies are spectrally encoded. Thus, the rescaled OCT signal *I*(*v*_*z*_) represents the modulated Fourier transform of the depth profile *F*(*z*) of the object:

3 $$I\left( {v_{z} } \right) \sim \int {F(z)\exp \left( { - i2\pi v_{z} z} \right)dz} .$$

Consequently, the depth profile *F*(*z*) can be reconstructed by the inverse Fourier transform of the rescaled OCT signal.

The width of the axial spatial frequency range is^26^:

4 $$\Delta \nu_{z} = \frac{2n\Delta \lambda }{{\lambda_{1} \lambda_{2} }},$$

where *λ*_1_,* λ*_2_ is the min and max wavelengths, *Δλ* is the spectral width, *Δλ* = *λ*_2_ −* λ*_1_. The spectral bandwidth for OCT is typically less than 200 nm, so the spatial frequency bandwidth is relatively narrow: for central wavelength *λ*_*c*_ = 1300 nm, *Δv*_*z*_ = 238 l/mm. The OCT image is formed using this small range of spatial frequencies; therefore, the quality of the OCT images is relatively poor in comparison with microscopy images. For example, in high-resolution microscopy, more than 1000 l/mm bandwidth of lateral spatial frequencies forms the image.

### Principle of detection and utilization of the high spatial frequency information from FDOCT signal

OCT usually works in reflection configuration, so the OCT signal is formed by light scattered at small, sub-wavelength size structures (Supplementary Fig. S1b). The coherent transfer function of the OCT is centred at zero lateral spatial frequencies but shifted to high axial spatial frequencies, which are larger than 1000 1/mm^27^. So, the OCT signal is formed by a relatively narrow bandwidth of the axial spatial frequencies, which is located in the high spatial frequency area of the Fourier domain.

In the FDOCT signal, each unique axial spatial frequency component of the object’s structure is encoded with its corresponding wavelength. This means that for each A-line acquired using FDOCT, complex amplitudes of the axial components of the 3D Fourier spectrum of the sample’s scattering potential are collected. Thus, the depth profile can be reconstructed via the inverse Fourier transform.

The limitations of conventional intensity-based OCT forbid access to submicron structural information and detection of nanoscale structural changes. However, even if it would be possible to get nanoscale resolution at a depth of about 1 mm within a scattering object, for example, in human skin, the reconstruction, say, of a 2 × 2 × 1 mm^3^ volume with nanoscale resolution would require a considerable amount of data. Spatial nano resolution is scientifically interesting, but for many applications, including early diagnostic of pathological processes, such resolution is unnecessary. Instead, it would be sufficient to detect nano-structural changes within some volumes of interests within the object. Indeed, given the data volumes, a bulk structural size parameter would be helpful in any case to store and visualize data in an efficient way.

One possible way to access information about high spatial frequencies is so called nano-sensitive OCT (nsOCT), (see “Materials and methods”). The nsOCT technique was invented to retain the high spatial frequency information and provide nano-sensitivity to structural changes^26,28–30^. Instead of using a conventional way to improve the resolution and sensitivity of the OCT by increasing the spectral bandwidth, nsOCT is a different approach, which permits the visualization of sub-wavelength structure from a single frame and provides nano-sensitivity to structural alteration at each volume of interest within a 3D biological object using the given spectral bandwidth.

Previously, it was shown that the axial Fourier spectrum of an object is very informative and highly sensitive to structural changes^17,21–25^. Following the SESF approach and having information about both **K**-space and reconstructed object space, we can perform structural characterization for a given volume of interest within the object with nanoscale sensitivity using a simple method utilizing the characteristics of the spatial frequency support in **K**-space. Namely, given that wavelength is spatially invariant (is not changed when translated from **K**-space to image space), by encoding each axial spatial frequency (or period) with one unique wavelength (Eq. 2), the spectrally encoded axial spatial frequency/period can be carried from the **K**-space to the reconstructed object space without compromising accuracy. By mapping the energy contribution of the spectrally encoded axial spatial period to each voxel within the 3D OCT image, we can perform structural characterization of every volume of interest of the object with nanoscale sensitivity.

Another group has used the nsOCT method to successfully detect the nanometer-scale structural changes of the human tympanic membrane in otitis media^31^. The related 3D SESF technique based on correlation of the axial spatial frequency profiles has also been published recently^32^.

### Experimental results

To confirm that information about small, sub-micron size structures in the object is present in FDOCT signal and for further validation of the nsOCT approach, we imaged two samples from OptiGrate Corp., USA, which consist of axial periodic structure with different periods. The corresponding periods of structure within the samples are 431.6 nm and 441.7 nm, sinusoidal refractive index variations 1.483 ± 0.001. These samples are, therefore, good representations of the single spatial frequencies. The samples were imaged using the SDOCT TELESTO III system from Thorlabs, Inc. with a central wavelength of 1300 nm and a depth resolution of 5.5 microns. Consequently, the structure within these samples cannot be resolved in the conventional OCT images produced from this system.

**Figure 1:**
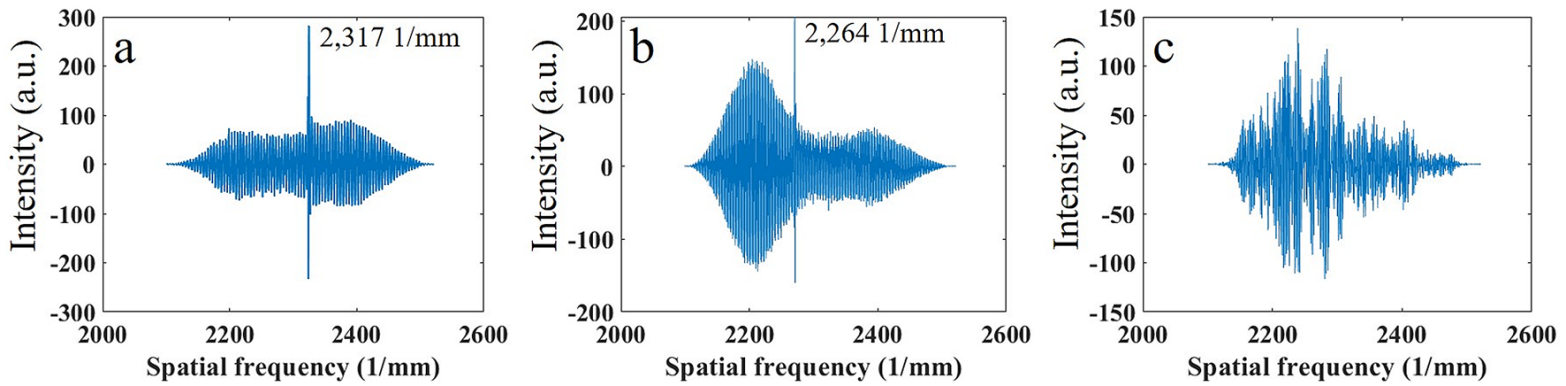
SDOCT signals for samples with (**a**) 431.6 nm period of structure, (**b**) 441.7 nm period of structure, and (**c**) for sample with random structure.

OCT signals (spectral interferograms) for these two samples, converted into axial spatial frequencies, are presented in Fig. 1. In Figs. 1a,b the peaks which correspond to structures with 431.6 nm and 441.7 nm periods (corresponding spatial frequencies are 2317 l/mm and 2264 l/mm) are clearly seen. These results confirm that high spatial frequencies that correspond to small, submicron size structure are present in the OCT signal. It should be noted that the spatial period of the structure can be calculated from these peaks, but the location of this structure within the sample is unknown. For comparison, the OCT signal from a random structure, Blu Tack (Bostik Industries), is shown in Fig. 1c, where many peaks are presented.

**Figure 2:**
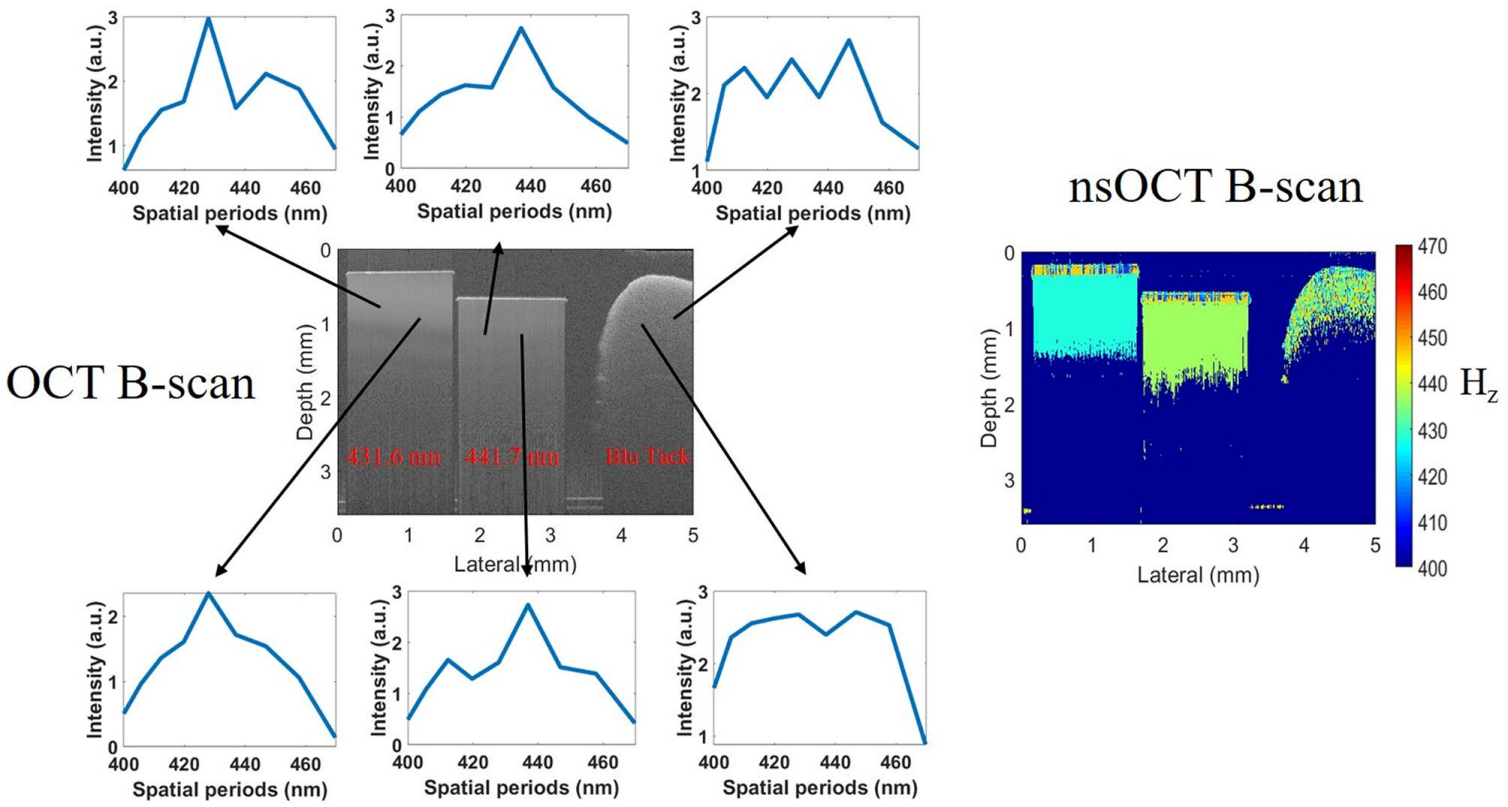
Reconstructed SDOCT and nsOCT images of two samples with axial periodic structures of 431.6 nm and 441.7 nm periods and a sample with random structure (BluTack) on the right. Reconstructed axial spatial period profiles for two points within each sample are shown. H_z_ in the color bar is the dominant axial spatial period in nm.

The corresponding reconstructed OCT and nsOCT images (B-frames) are presented in Fig. 2. From conventional OCT images, it is impossible to detect differences in the structure between the samples. Information about the sub-wavelength structure presented in the OCT signal is lost, as explained in previous sections. Using the nsOCT approach, we reconstructed axial spatial period profiles, where information about the small sub-wavelength structure is present, for each point within the images. Examples of such profiles for two different points within each sample are presented in Fig. 2. For two samples with periodic structure, these profiles have peaks that correspond to the dominant period of structure at given locations within the sample. Namely, for the left sample, peaks are near 431 nm, and for the middle sample, peaks near 441 nm spatial periods correspond to actual periods of the structure. These results demonstrate that the signal from a small, sub-wavelength size structure is translated to the image domain and present in the axial spatial frequency profiles reconstructed for each volume of interest within the image. There are no clear single peaks in the profiles for the sample on the right, which comprises primarily random structures. The nsOCT image in Fig. 2 is formed as a color map of the dominant period of the structure. In this image, in contrast to the conventional OCT image, we can see samples with different spatial periods of structures: two samples with uniform structure (the same color in different locations within each sample), where the structure has the same dominant period at different locations, and the sample with the random structure on the right, where the structure has different dominant periods at different locations within the sample. The difference of 10 nm in spatial periods of structure between samples, which represent single spatial harmonics of the structure, can be clearly detected using the nsOCT approach.

For biological tissues, we have continuous profiles which consist of many different spatial frequencies. Any nano-structural changes in spatial harmonics within scattering objects will change the spatial frequency profiles, as we can see in experiments with phantoms (Fig. 2), and so can be detected. To demonstrate the potential of nsOCT for biomedical applications, we performed imaging of mesenchymal stem cells (MSCs) induced to undergo chondrogenic differentiation, the process by which cartilage is formed from condensed mesenchyme tissue^33^. Induction of MSC chondrogenesis ex vivo involves exposure of cells to transforming growth factor (TGF)-β3 in micromass culture under low oxygen conditions, to mimic the natural hypoxic, 3-D environment of cartilage. The process of chondrogenesis occurs in two separate phases: mesenchymal condensation and subsequent differentiation into chondrocytes through defined stages^34^. The formation of a precartilage condensation matrix, rich in fibronectin and collagen I is evident in the early stages of the process. A transition to a collagen II- and aggrecan-rich cartilage matrix is then observed^35^. The structure and organization of cells and extracellular matrix (ECM) changes as cells progress through the different stages of this process. For example, between day 1 and day 4, changes in structure occur (Supplementary Figs. S2–S4 in Supplementary information) that are associated with critical molecular events associated with commitment to differentiation, e.g. a required proliferation step before expression of cartilage-specific type II collagen is initiated at about day 3. We investigated if nsOCT was capable of detecting these structural changes associated with the molecular events occurring by imaging cells (n = 3 biological replicates) at different time points throughout the process (Day 1, 4 and 21). An undifferentiated control for all donors was included and maintained in culture for 21 days as a negative control. The capacity of nsOCT to detect structural differences over the course of the differentiation process was assessed with donor variability associated with the progression of this assay evaluated between donors by histological analysis of corresponding chondrogenic and negative control pellets. The color bar in the nsOCT images represent the dominant axial optical spatial period of the structure in nm.

**Figure 3:**
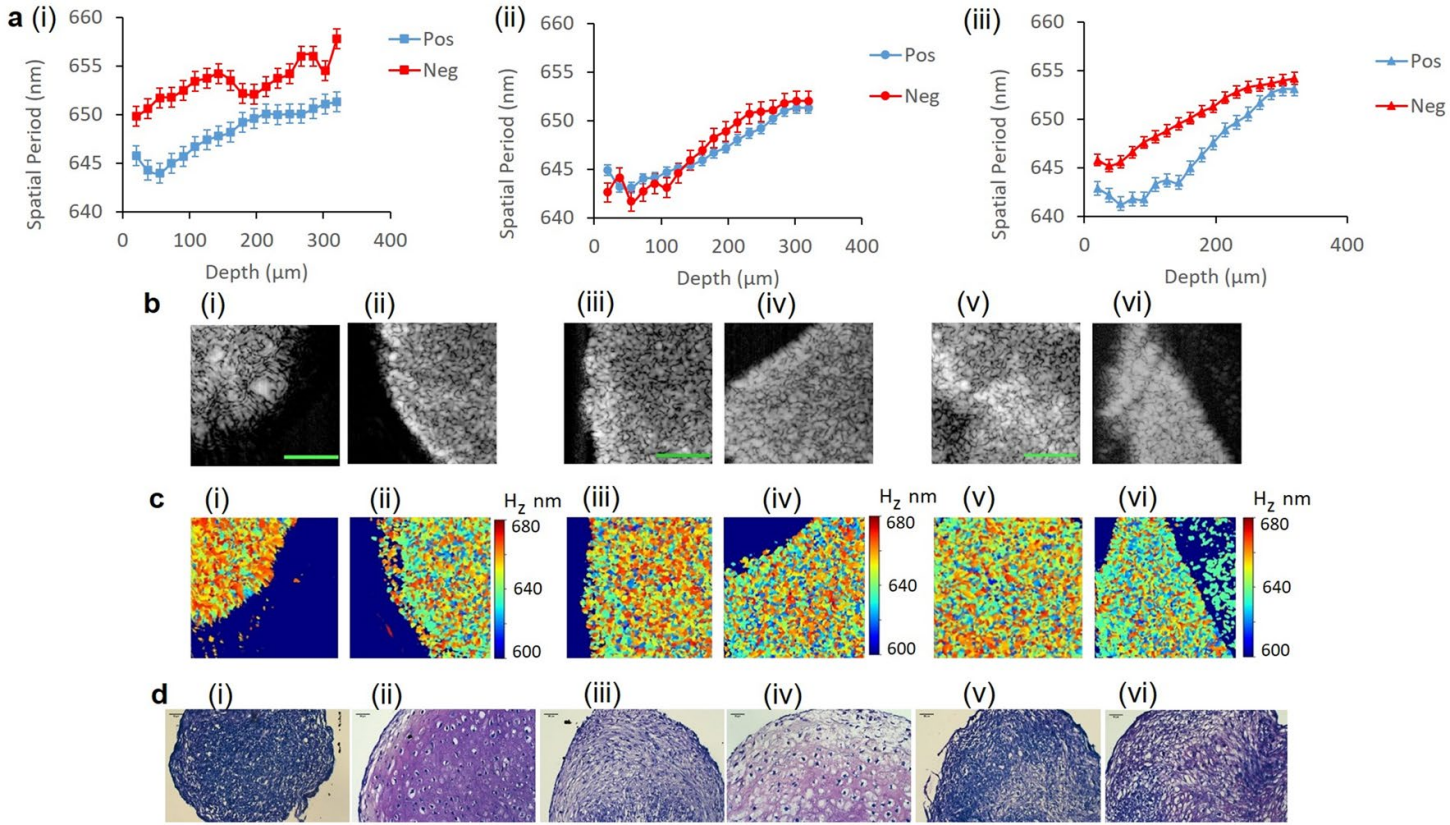
Conventional SDOCT, nsOCT imaging and histological analysis of day 21 chondrogenic and undifferentiated MSC pellets from three biological replicates. (**a**) The dominant axial spatial period profiles (nm) versus depth (µm) of day 21 positive cell pellets and negative controls from three donors. (**b**) Conventional SDOCT, (**c**) nsOCT enface images and (**d**) Histological analysis and toluidine blue staining of MSC pellets for Donor 1 negative (i) and positive (ii) samples at depth 90 μm, Donor 2 negative (iii) and positive (iv) samples at depth 170 μm and Donor 3 negative (v) and positive (vi) samples at depth 60 μm. The color bar presents the dominant spatial period of the structure H_z_ in nm. The scale bar is 0.1 mm for OCT and nsOCT images, and = 50 µm for histological images.

Conventional SDOCT, nsOCT imaging and histological analysis of day 21 chondrogenic and undifferentiated MSC pellets from three biological replicates are presented in Fig. 3a–d. For all biological replicates/donors, nsOCT imaging detected a decrease in the mean spatial period of the structure of positive cultures (day 21), suggesting a decrease in the dominant structure compared to undifferentiated or negative controls (Fig. 3a (i)–(iii)). Examples of the enface nsOCT images, formed as color maps of the dominant spatial period of the structure, are presented in Fig. 3b(i)–(vi)). The difference in the mean spatial period between positive and negative cultures between donors also varied. For example, a difference of 5.7 nm and 8.2 nm, respectively, in the mean spatial period of the dominant structure was observed for Donor 1 (Fig. 3a(i) and Supplementary Fig. S5) and Donor 3 (Fig. 3a(iii) and Supplementary Fig. S7). The larger difference in the mean spatial periods correlated with the enhanced chondrogenic capacity demonstrated by Donor 1 cells.

Moreover, a difference of just 3.1 nm between positive and negative cultures was detected for Donor 2 (Fig. 3a(ii)), indicating only a minor difference in structure between these samples. This observation was validated by histological staining with toluidine blue. The presence of some positive metachromasia, represented by decreased intensity of blue stain (Fig. 3d(i and v)) and a hint of characteristic pink/purple stain indicating production of cartilage-specific proteoglycans in negative cultures (Fig. 3d(iii)) indicated an innate capacity for chondrogenesis in these donor cells with the usual requirement for a growth factor stimulus not required, although differences in the extent of differentiation was less without exposure to TGF-β3.

**Figure 4:**
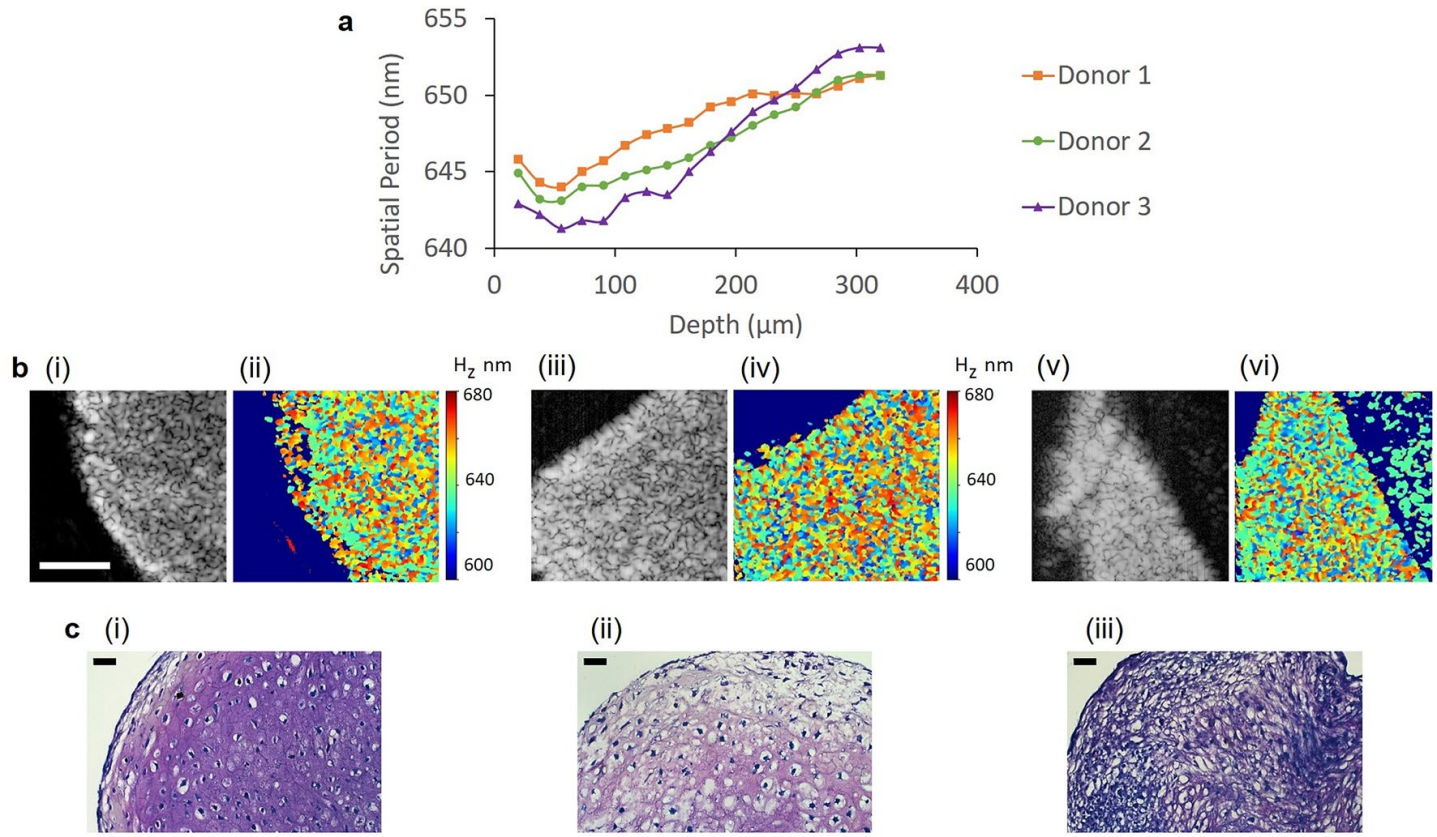
Conventional OCT, nsOCT imaging and histological analysis of day 21 chondrogenic MSC cell pellets from three donors. (**a**) The dominant axial spatial period profiles (nm) versus depth (µm) of day 21 positive cell pellets from three donors. (**b**) Conventional enface SDOCT and nsOCT images of Donor 1 (i), (ii), Donor 2 (iii), (iv) and Donor 3 (v), (vi) day 21 pellets. (**c**) Histological analysis and toluidine blue staining of MSC pellets for Donor 1 (i), Donor 2 (ii) and Donor 3 (iii). Color bar presents the dominant spatial period of the structure H_z_ in nm. Scale bar is 0.1 mm for OCT and nsOCT images and 50 µm for histological images.

Additionally, nsOCT was capable of detecting differences in the extent of chondrogenesis between positive chondrogenic pellets. Donor 1 demonstrated significant uniform proteoglycan deposition in the matrix surrounding a relatively large cell body, evidence of terminal differentiation and chondrocyte hypertrophy. Toluidine blue staining, which preferentially stains for aggrecan^36^, the primary proteoglycan present in cartilaginous structures, indicated the production of a mature cartilage phenotype in this sample (Fig. 4c(i)) in all but the surface layer where undifferentiated MSC-like cells are unstained. Corresponding nsOCT data demonstrated an initial reduction in the mean spatial period of the dominant size in this depth area from 20 to 60 µm (Fig. 4a). From approximately 60 µm, the mean spatial period increased in this sample, suggesting increased proteoglycan deposition with increasing depth associated with the structural organization of the pellet and mature cartilage production.

These nanoscale structural changes with increasing depth were not detectable using conventional OCT (Fig. 4b(i)). The second donor assessed performed differently and demonstrated a lower capacity for chondrogenesis and ECM production (Fig. 4c(ii)). The nsOCT data demonstrated reduced mean spatial periods throughout differentiation in comparison to data obtained using Donor 1 (Fig. 4a). This sample also followed a similar trend in that the outer regions of the pellet had a depleted matrix with a disorganized structure as represented by a smaller dominant size. However, with increasing depths throughout the pellet, the dominant structural size increased with proteoglycan deposition and ECM production (Fig. 4a,b (iv)). The third donor used in this assessment showed the least propensity to produce ECM, as shown by a reduction in positive metachromatic staining (Fig. 4c(iii)) and the lowest mean spatial period in nsOCT imaging and analysis (Fig. 4a). Additional results on samples at different days and positive and negative samples are presented in the Supplementary Information.

## Discussion and conclusion

The formation of the FDOCT signal has been considered using general scattering theory. We demonstrated that:

- detected FDOCT spectral interference signal corresponds to axial components of the 3D Fourier spectrum of the limited bandwidth of high axial spatial frequency.
- information about high spatial frequencies of the object, which correspond to small, sub-wavelength size period of the spatial harmonic of the structure, presents in the OCT signal.

It was shown that the high spatial frequency information, which is discarded in conventional OCT, can be utilized by applying the nsOCT approach. In contrast to known methods to detect structural changes with high sensitivity by comparison images recorded at different time moments, like phase-sensitive OCT^13^, the nsOCT visualizes the sub-wavelength structure from a single image. The theoretical basis for the nsOCT approach was presented and validated using samples with known sub-micron internal structure. We show theoretically and experimentally that information about small sub-wavelength size structure presents in the OCT signal and that this information can be translated into the image domain as the high axial spatial frequency profiles, reconstructed for each pixel within the image.

For the realization of the nsOCT method, the spectral interference OCT signal is used. Using the detected FDOCT signal, it is possible to reconstruct OCT images of the strongly scattering media, and nsOCT can then be applied to map high spatial frequency information within each location in the reconstructed FDOCT image. Therefore, the nsOCT approach can be applied to strongly scattering media, similar to conventional OCT, if we have the corresponding spectral interference signal from these media. Like conventional OCT, the nsOCT image formation is based on scattering theory and does not consider absorption.

Most practical OCT systems can be approximated using a normal incident illumination beam, and Eq. (2) is sufficient. However, for large NA objective lenses, Eq. (2) is not valid anymore, and Eq. (1) is more accurate. That means due to the increased NA of a lens, the one-to-one correspondence between wavelengths and axial spatial frequencies reduces, and the complex Fourier spectrum of axial spatial frequencies will not adequately formed anymore. Consequently, to avoid the computation of an erroneous depth profile, it becomes essential to apply Eq. (1) for accurate image reconstruction. The rigorous relationship with a focused beam illumination in OCT is described in Ref.^12^, which is especially important for realizing optical coherence microscopy where objective lenses with large NA are used.

The main limitation of the nsOCT technique is the limited spectral bandwidth, which also limits the image quality and resolution of conventional OCT images. OCT detects from all the axial components of the 3D Fourier spectrum, scattered from the object, only a limited bandwidth of spatial frequencies. As demonstrated with nsOCT, even using a limited bandwidth of spatial frequencies, it is possible to visualize structural changes in biological tissues and other objects. The use of broadband illumination would increase the detected spatial frequency bandwidth along with the axial resolution of the nsOCT and conventional OCT images.

Quantitative depth-resolved visualization of the structure at clinically relevant depths and within the detected range of high spatial frequencies with nano-sensitivity has been shown. Differences in structural periods as small as 10 nm has been detected using just a single frame. The ability of nsOCT to visualize sub-wavelength structures from a single frame creates the potential to monitor nanoscale structural changes in real-time.

The potential of nsOCT for visualization and detection of structural changes at the nanoscale in biological samples was demonstrated. The process of stem cell chondrogenic differentiation occurs in two separate phases: mesenchymal condensation followed by chondrocyte differentiation^37^. In vitro, the formation of a 3D structure following pelleting of MSCs mimics the early phase of mesenchymal condensation. Critical changes that occur in MSCs during this phase, were observed using nsOCT imaging as an increase in the dominant size of the structures, observed between day 1 and day 4 (Supplementary Figs. S2–S4 in supplementary information). A lower mean spatial period of samples at lower depths is indicative of undifferentiated cells, where typically cells found at the periphery of the pellet remain in an undifferentiated state. As MSC chondrocyte differentiation progresses, many changes occur to both the cells and the matrix they elaborate. These changes were detectable using nsOCT imaging as a reduction in the mean spatial period which is the local maximum of the spatial frequency component of the scattering potential that has entered the detection band. As such, nsOCT imaging was capable of distinguishing between MSC cell pellets that were induced to undergo chondrogenesis and produce cartilage before undergoing terminal differentiation and hypertrophy and MSC cell pellets that were not induced to differentiate (Fig. 4) and (Supplementary Figs. S5–S7 in Supplementary Information). The capacity of nsOCT to detect these changes at the nanoscale level suggests that this technique can differentiate between healthy, normal articular cartilage and that which has been degraded and depleted of the matrix as seen in osteoarthritic tissue. Future work will assess the capacity of nsOCT to monitor changes in chondrogenic cell pellets that have been treated with inflammatory mediators using an ex vivo model for osteoarthritis and during the development of disease using mouse models^38^.

The presented theoretical and experimental results provide a strong basis for the nsOCT approach and permit to extend the possibility of OCT imaging into the sub-wavelength range with nano-sensitivity to structural changes at clinically relevant depths. It is expected that nsOCT can lead to new application areas and increase the potential to study pathological processes and enable new methods for early diagnostics.

## Materials and methods

### Experimental setup

A commercial SDOCT system, Telesto III, Thorlabs, Inc. New Jersey, United States, was used for the data acquisition. The central wavelength is 1300 nm, axial resolution 5.5 μm, imaging depth 3.6 mm in air. Sensitivity is 96 dB at 76 kHz rate. Objective lens LSM03, NA = 0.055, lateral resolution = 13 μm was used in our experiments. The number of sampling points (pixels) for detected OCT signals was 2048, which corresponds to 1024 points in the reconstructed image. The number of pixels in lateral directions for images in Figs. 3 and 4 is 300 × 300.

### Image formation and processing

**Figure 5:**
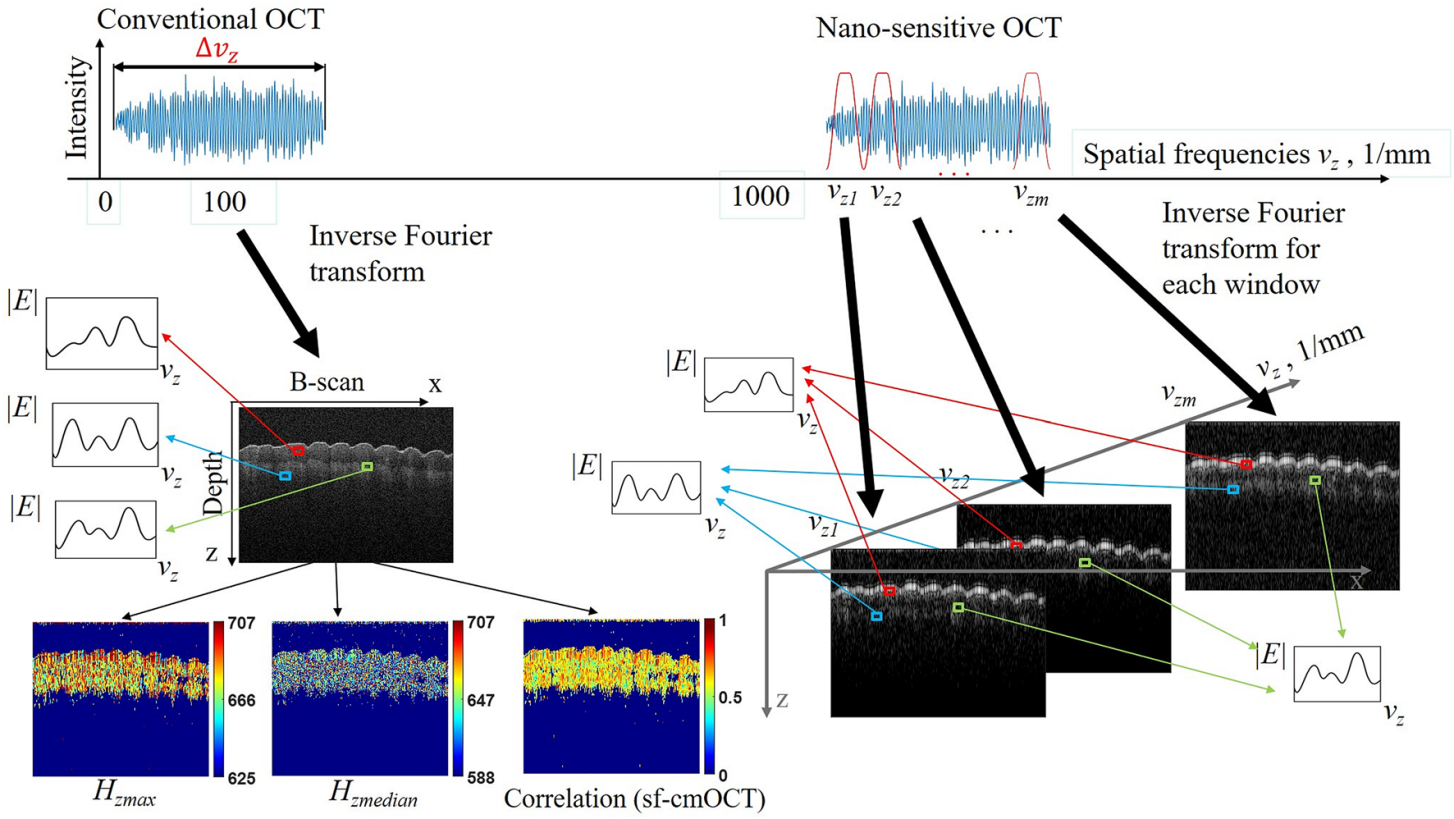
Principle of detection of the high spatial frequency information from FDOCT signal and incorporation in conventional OCT image.

A schematic of the principle of detecting the high spatial frequency information from FDOCT signal and incorporation in conventional OCT images (nsOCT approach) is presented in Fig. 5. For nsOCT processing, the collected complex amplitudes of the spectrum are converted to complex amplitudes of the axial spatial frequencies. As described above (Supplementary Fig. S1 and Eq. (2)), it can be done by noting that each wavelength defines a unique Ewald sphere cap in **K**-space, which is transformed into the point on the axis for a single A-scan. The complex spectrum of axial spatial frequencies is placed in the corresponding region of high spatial frequencies in the Fourier domain *v*_*zmin*_ = *2n/λ*_*max*_ to *v*_*zmax*_ = *2n/λ*_*min*_ (Fig. 5). In contrast, conventional FDOCT works as if the spectrum is placed in the region of low spatial frequencies from *v*_*zmin*_ = *2n/d* to *v*_*zmax*_ = *2n/Δλ* where *d* is the penetration depth. After the inverse Fourier transform of the OCT signal, the depth profile can be reconstructed, and corresponding B or C-scans can be formed using the appropriate number of A-lines.

In the nsOCT approach, the complex spectrum of axial spatial frequencies, placed in the proper region of high spatial frequencies, can be decomposed into multiple sub-bands. The contribution of each *m*th sub-band *Δv*_*zm*_ = *v*_*zm*+1_ − *v*_*zm*_ into each point in a depth profile can be described by the inverse Fourier transform:

7 $$F_{m} (z) \sim \frac{1}{2\pi }\int_{{v_{zm} }}^{{v_{zm + 1} }} {I(v_{z} )\exp (i2\pi zv_{z} )dv_{z} } .$$

We can also divide the object space into multiple volumes of interest (VOI) and calculate such energy contribution from *m*th spatial frequency bandwidth for each VOI of the 3D OCT image. Similarly, the contribution of all spatial frequency sub-bands into each VOI can be calculated. Using this procedure, the axial spatial frequency or period profiles can be reconstructed for each pixel of the 2D image (B-scan), as shown in Fig. 5, or for each pixel of the 2D enface image or for each voxel of the 3D image (C-scan). So, besides the low spatial frequency bandwidth in conventional OCT, we utilize the high frequency content which is present in the OCT signal. As a result, the sub-wavelength spatial periods of the local structure are detected, and nanoscale structural alterations within each voxel can be visualized.

Thus, to form a nsOCT image we

- Re-scale the spectral interference signal after pre-processing into spatial frequencies. The wavelength axis is converted to spatial frequency axis using Eq. (2) in the manuscript.
- Decompose spectrum with a Tukey window (other windows also can be used) into multiple sub-bands. The Tukey windows are zero-padded to match the full-length spectral array. The spectral window position corresponds to a particular spatial frequency related to the bandwidth of the used light source.
- Apply the Fast Fourier-Transforming (FFT) to each selected spectrum within sub-bands to get the A-scan (depth profile). Each A-scan is formed by a limited bandwidth of the spatial frequencies from each sub-band and corresponds to a particular spatial frequency.
- Calculate the energy contribution from each sub-band into the distribution of spatial periods at each depth within each A-line and reconstruct the axial spatial frequency or period profiles at each pixel or voxel of the image. The spatial period is the reciprocal of spatial frequency.
- Form the nsOCT image as a color map of the dominant spatial period distribution, which is the spatial period at the max signal, as is shown in Fig. 5. These spatial periods are dominant within the detected bandwidth of spatial frequencies (periods).

The nsOCT images can also be formed as color maps of other informative parameters of the axial spatial frequency profiles, for example as a color map of the median spatial frequency, as a color map of the correlation between profiles, or any other mathematical evaluation. Color bars provide quantitative information about structure at each volume of interest.

### Samples preparation

To demonstrate that information about the small, sub-wavelength size structure of the object is presented in FDOCT signal and for further validation of the nsOCT approach, we used the two samples from OptiGrate Corp., USA. These samples consist of axial periodic structures with different well-known periods. The corresponding periods of structure within samples are 431.6 nm and 441.7 nm, sinusoidal refractive index variations 1.483 ± 0.001.

The BluTack sample was just a 3D piece made of BluTack purchased from (Bostik Industries).

#### Preparation of MSC cell pellets and histological analysis

Human MSCs (hMSCs) were isolated following bone marrow aspiration from the iliac crest of healthy donors. Informed consent from patients and approval from the Clinical Research Ethical Committee at University College Hospital, Galway, Ireland, was obtained before all procedures were performed. MSCs were isolated from bone marrow based on plastic adherence in culture and expanded in alpha-Minimum Essential Medium supplemented with 10 foetal bovine serum, 1% penicillin/streptomycin, and one ng/ml recombinant human basic fibroblast growth factor. MSCs expanded to passage three were induced to undergo chondrogenic differentiation using micromass 3-D pellet culture at 2% O2 and 10 ng/ml transforming growth factor (TGF)-β3 as described previously^39^. All cell pellets contained 2.5 × 105 cells and were maintained in culture for either one, four or twenty-one days, followed by fixation in 10% neutral buffered formalin. Pellets used for nsOCT imaging were dehydrated through an alcohol gradient (70%, 90%, and 100% for 1 h each) to prevent changes in the hydration states of pellets interfering with imaging. For histological analysis, pellets were processed automatically using a tissue processor (cycles of 70% IMS for one hour, 90% IMS for one hour, 100% IMS for three hours, xylene for three hours and melted paraffin wax for three hours), and embedded in paraffin wax followed by sectioning into five µM cross-sections and mounted onto slides for staining. Each pellet was stained with 0.5% toluidine blue solution and imaged using the Olympus 1X71 microscope. According to absorbance plot for collagen II presented in Fig. 2 in Ref.^40^, absorbance is significantly reduced versus wavelength and at 700 nm is consistently low, suggesting that changes, detected by nsOCT are primarily changes of the of the tissue structure.

### Ethical approval

All methods were carried out in accordance with relevant guidelines and regulations.

## Supplementary Information

Supplementary Information.

## Data Availability

The data sets generated and analyzed during the current study are available from the corresponding author on reasonable request.
