# Supplementary material for: Accessing depth-resolved high spatial frequency content from the optical coherence tomography signal
Source: Sci Rep. 2021 Aug 24;11:17123. doi: 10.1038/s41598-021-96619-7 (PMC8385072; doi:10.1038/s41598-021-96619-7)
Supplement: Supplementary file 1 — Supplementary Information. [file 41598_2021_96619_MOESM1_ESM.pdf]

SUPPLEMENTARY INFORMATION

Accessing depth resolved high spatial frequency content from the optical coherence tomography signal

Sergey Alexandrov,[1*] Anand Arangath,[1] Yi Zhou,[1] Mary Murphy,[2] Niamh Duffy[2], Kai Neuhaus[1], Georgina Shaw[2], Ryan McAuley[1], and Martin Leahy[1]

[1]*National University of Ireland, National Biophotonics and Imaging Platform, School of Physics, Tissue Optics and Microcirculation Imaging Group. Galway, H91 TK33 Ireland.*

[2]*Regenerative Medicine Institute, National University of Ireland Galway, H91 W2TY, Ireland*

**Corresponding author*: Sergey Alexandrov sergey.alexandrov@nuigalway.ie, phone 353 91 49 5350, fax: 353 91495529

Contents

Theoretical analysis of the scattered field in reflection configuration.

Figure S1. Spatial frequency representation in K-space.

Supplementary Figures S2 – S4 for samples at day 1 and day 4.

Supplementary Figures S5 – S7 for positive and negative samples.

Theoretical analysis of the scattering optical wave in reflection configuration

It is well known that the object's structure can be described using 3D function, which is usually called the scattering potential[16]:

$$F(\mathbf{r}) = \frac{1}{4\pi} k_0^{\,2} \left[n^2(\mathbf{r}) - 1 \right], \qquad \text{(S1)}$$

or its 3D Fourier transform:

$$F(\mathbf{K}) = \int F(\mathbf{r}) \exp\left(-i\mathbf{K}\bullet\mathbf{r}\right) d^3 r, \qquad \text{(S2)}$$

where n – refractive index, **K** is the spatial frequency vector.

We restrict our consideration to the first Born approximation, do not show the dependence on time and ignore polarization effects. Illumination wave under the scalar representation could be written as

$$U_i(\mathbf{r}, k) = U_0(k) \exp\left[i\left(k_{0x} x + k_{0y} y - k_{0z} z\right)\right], \qquad \text{(S3)}$$

Where $k=2\pi/\lambda$ – wavenumber, $k_{0x}=2\pi\nu_x$, $k_{0y}=2\pi\nu_y$, $k_{0z}=2\pi\nu_{0z}$, ν_{0x}, ν_{0y}, ν_{0z} – are the incident spatial frequencies, x, y and z are cartesian coordinates of a point $\boldsymbol{r}$ within the object space.

The scattered wave $U_S(\mathbf{r}`)$ at some point **r`** can be written as a volume integral[16,17]:

$$U_s\left(\mathbf{r}'\right) = \int_V F(\mathbf{r}) U_i(\mathbf{r}, k) G\left(\mathbf{r}' - \mathbf{r}, k\right) d\mathbf{r}, \qquad \text{(S4)}$$

Where $G\left(\mathbf{r}' - \mathbf{r}, k\right)$ represents the Green function:

$$G\left(\mathbf{r}' - \mathbf{r}, k\right) = \frac{\exp\left(ik\left|\mathbf{r}' - \mathbf{r}\right|\right)}{\left|\mathbf{r}' - \mathbf{r}\right|}, \qquad \text{(S5)}$$

Under the Born approximation, we can use Ui instead of $U = Ui + Us$ to describe the scattered wave in Eq. (S4).

After some simplifications we get:

$$U_s(\mathbf{r}') = \frac{i}{2\pi} \iint_\infty \tilde{F}\left(k_x - k_{0x}, k_y - k_{0y}, k_z + k_{0z}\right) \exp\left(i\mathbf{k}\cdot\mathbf{r}'\right) dk_x dk_y, \qquad \text{(S6)}$$

where

$$\tilde{F}(\mathbf{K}) = \tilde{F}\left(k_x - k_{0x}, k_y - k_{0y}, k_z + k_{0z}\right)$$
$$= U_0(k) \int_Z \frac{1}{k_z} \hat{F}\left(k_x, k_y, z, k_{0x}, k_{0y}\right) \exp[-i(k_{0z} + k_z)z] dz' \qquad \text{(S7)}$$

$\tilde{F}$ is the angular spectrum[18] of the complex wave scattered by the lateral cross-section of the object located at depth z. The angular spectrum is given by 2D Fourier transform of the scattering potential Eq. (S1). The integral in Eq. (S7) represents the superposition of the angular spectrum of the complex backscattered waves centred at (k_{0x}, k_{0y}) from all depths within the object. So, this equation provides information about the entire 3D structure of the object. The structure is described by spatial frequency vector, which can be written as:

$$\mathbf{K}=\mathbf{k}_s-\mathbf{k}_0=\frac{2\pi n}{\lambda}\left(\mathbf{s}-\mathbf{s}_0\right)=2\pi n\left[\left(v_x\mathbf{i}+v_y\mathbf{j}+v_z\mathbf{k}\right)-\left(v_{0x}\mathbf{i}+v_{0y}\mathbf{j}-v_{0z}\mathbf{k}\right)\right], \quad \text{(S8)}$$

where $\boldsymbol{s}$, $\boldsymbol{s}_0$ are unit vectors of scattered and illumination waves (Fig. S1a), v_x, v_y and v_z – spatial frequencies of the object's structure along Cartesian coordinates.

Equation (S8) shows that the complex amplitude of the scattered wave at a given wavelength in the far zone for a given direction depends entirely on only one Fourier component (one spatial frequency) of the 3D scattering potential, labelled by the vector **K**. At a constant illumination angle, the end point of each vector Fourier component of the 3D scattering potential for given collection angle corresponds to a point on Ewald's sphere. Illumination and collection geometry is presented in Fig. S1a. If the object is illuminated by a plane wave with a certain spectral bandwidth, then the spatial frequencies distribution in **K**-space for all collection angles can be illustrated as multiple Ewald's spheres with different diameters. For an illumination beam at normal incidence and n = 1 the back scattered wave Eq. (S7) gives the 3D frequency distribution in **K**-space. Example of such distribution in 2D plane for spectral bandwidth 1220 nm – 1400 nm is presented in Fig. S1b.

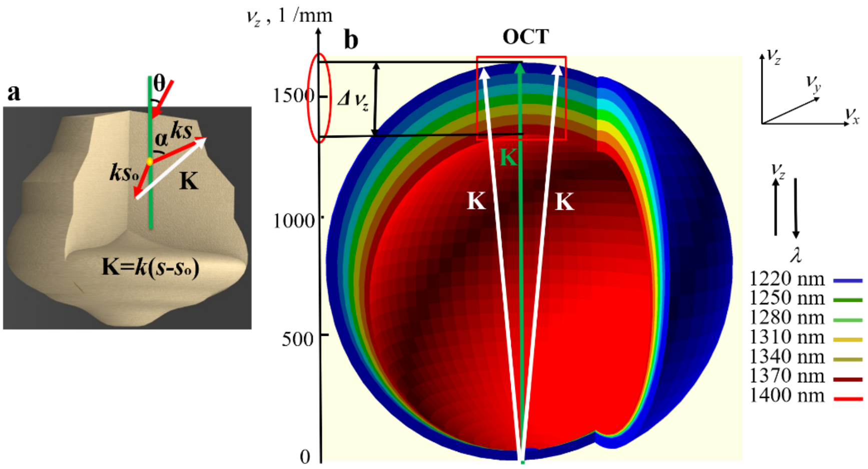

Figure S1. Spatial frequency representation in K-space. **(a)** – Schematic of object illumination and collection; **(b)** – spatial frequency representation in **K**-space depending on wavelength.

If we detect the scattered field in **K**-space (the complex amplitudes of all Fourier components), then we could synthesize the 3D Fourier transform of the scattering potential Eq. (S2). After that the scattering potential can be reconstructed via 3D inverse Fourier transform. However, even if all spatial frequencies will be captured, the scattering potential

will be reconstructed under low-pass filtered approximation and the best possible resolution will be about half of wavelength[16]. The objective lens, used for collection, will further limit the accessible bandwidth of the spatial frequencies, depending on the numerical aperture (NA), and instead of spheres we will have NA- restricted Ewald sphere caps as it shown in Fig. S1b within the red rectangle.

Supplementary Figures S2 – S4 for samples at day 1 and day 4.

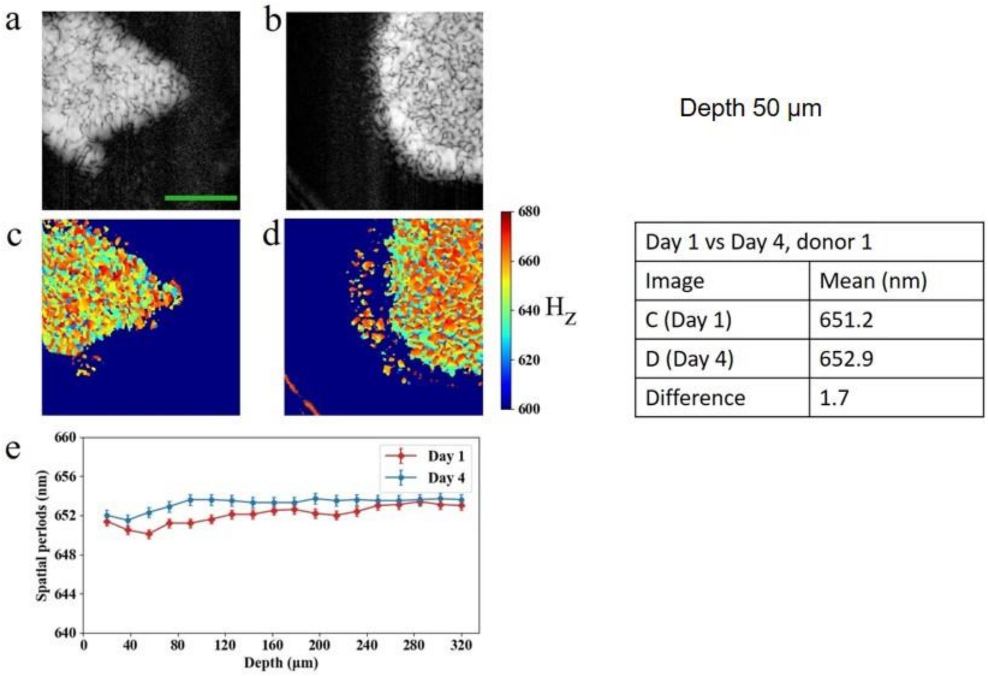

Day 1 vs Day 4, donor 1	
Image	Mean (nm)
C (Day 1)	651.2
D (Day 4)	652.9
Difference	1.7

Fig. S2. Conventional OCT en face a,b and corresponding nsOCT c,d images of MSC from donor 1 that have been induced to undergo chondrogenic differentiation for one **a**,**c** or four **b**,**d** days in micromass culture; **e** – averaged along images the dominant axial spatial period profiles versus depth, with standard deviations, red – for day 1 and blue – for day 4. H_z – is the dominant axial optical spatial period of the structure in nm. The scale bar (green line) is 0.1 mm.

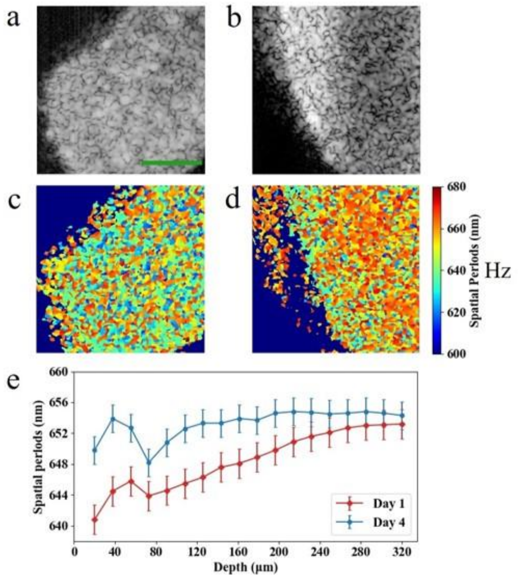

Depth 170 µm

Day 1 vs Day 4, donor 2	
Image	Mean (nm)
C (Day 1)	645.8
D (Day 4)	653.4
Difference	7.6

Fig. S3. Conventional OCT en face a,b and corresponding nsOCT c,d images of MSC from donor 2 that have been induced to undergo chondrogenic differentiation for one **a**,**c** or four **b**,**d** days in micromass culture; **e** – averaged along images the dominant axial spatial period profiles versus depth, with standard deviations, red – for day 1 and blue – for day 4. H_z – is the dominant axial optical spatial period of the structure in nm. The scale bar (green line) is 0.1 mm.

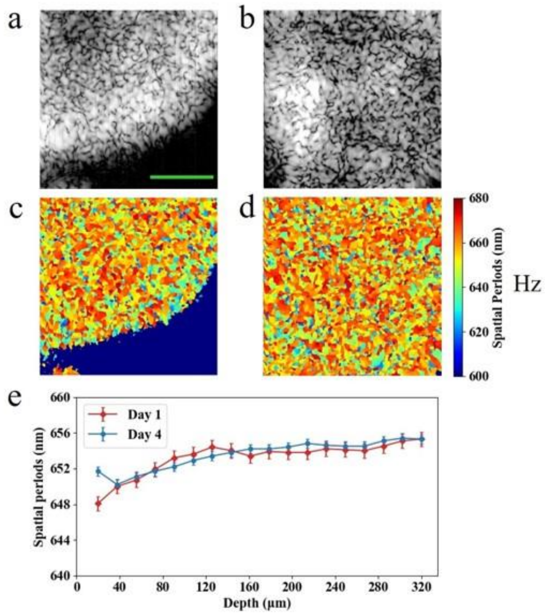

Depth 90 µm

Day 1 vs Day 4, donor 3	
Image	Mean (nm)
C (Day 1)	654.11
D (Day 4)	655.23
Difference =	1.12

Fig. S4. Conventional OCT en face a,b and corresponding nsOCT c,d images of MSC from donor 3 that have been induced to undergo chondrogenic differentiation for one **a**,**c** or four **b**,**d** days in micromass culture; **e** – averaged along images the dominant axial spatial period profiles versus depth, with standard deviations, red – for day 1 and blue – for day 4. H_z – is the dominant axial optical spatial period of the structure in nm. The scale bar (green line) is 0.1 mm.

Supplementary Figures S5 – S7 for positive and negative samples.

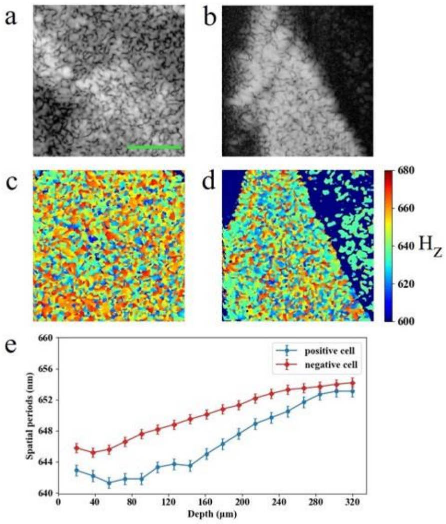

Depth 60 μm

Negative vs Positive donor 1	
Image	Mean (nm)
C (negative)	647.2
D (positive)	641.5
Difference	5.7

Fig. S5. Conventional OCT en face images a,b and corresponding nsOCT c,d images from donor 1 of **a,c** - chondrogenic pellets from MSC not exposed to TGF-β3 for the same period of time as a negative control. **b,d** - MSCs treated with TGF-β3 for 21 days in pellet culture to induce chondrogenic differentiation of MSCs. **e** – averaged dominant axial optical spatial period profiles versus depth with standard deviations, red – for negative cells and blue – for positive cells. H_z – is the dominant axial optical spatial period of the structure in nm. The scale bar is 0.1 mm.

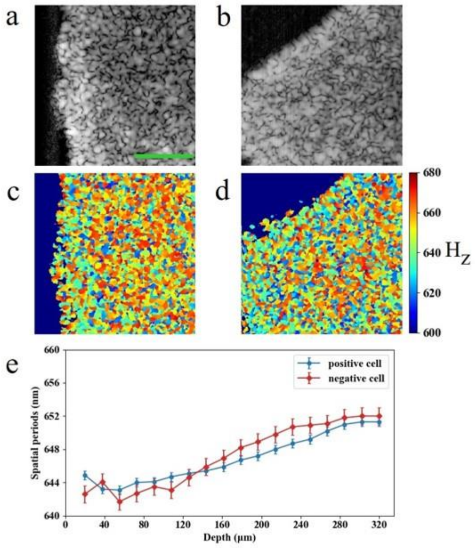

Depth 170 μm

Negative vs Positive donor 2	
Image	Mean (nm)
C (negative)	648.3
D (positive)	645.2
Difference	3.1

Fig. S6. Conventional OCT en face images a,b and corresponding nsOCT c,d images from donor 2 of **a,c** - chondrogenic pellets from MSC not exposed to TGF-β3 for the same period of time as a negative control. **b,d** - MSCs treated with TGF-β3 for 21 days in pellet culture to induce chondrogenic differentiation of MSCs. **e** – averaged dominant axial optical spatial period profiles versus depth with standard deviations, red – for negative cells and blue – for positive cells. H_z – is the dominant axial optical spatial period of the structure in nm. The scale bar is 0.1 mm.

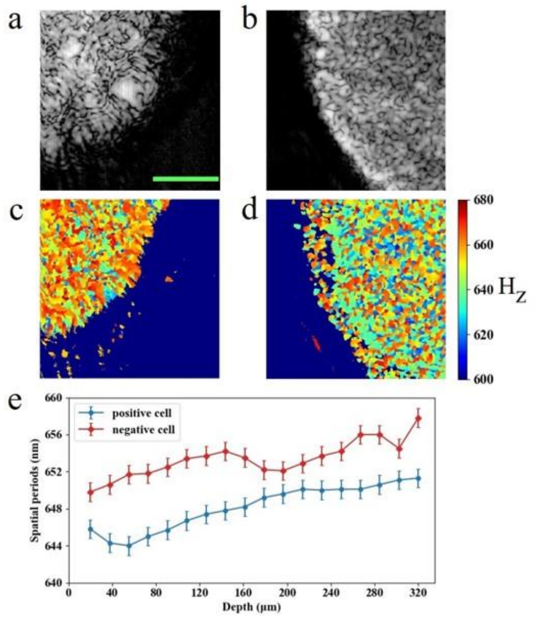

Depth 90 µm

Negative vs Positive, donor 3	
Image	Mean (nm)
C (negative)	654.1
D (positive)	645.9
Difference	8.2

Fig. S7. Conventional OCT en face images a,b and corresponding nsOCT c,d images from donor 3 of **a,c** - chondrogenic pellets from MSC not exposed to TGF-β3 for the same period of time as a negative control. **b,d** - MSCs treated with TGF-β3 for 21 days in pellet culture to induce chondrogenic differentiation of MSCs. **e** – averaged dominant axial optical spatial period profiles versus depth with standard deviations, red – for negative cells and blue – for positive cells. H_z – is the dominant axial optical spatial period of the structure in nm. The scale bar is 0.1 mm.
